# Patient prioritization tools and their effectiveness in non-emergency healthcare services: a systematic review protocol

**DOI:** 10.1186/s13643-019-0992-x

**Published:** 2019-03-30

**Authors:** Julien Déry, Angel Ruiz, François Routhier, Marie-Pierre Gagnon, André Côté, Daoud Ait-Kadi, Valérie Bélanger, Simon Deslauriers, Marie-Eve Lamontagne

**Affiliations:** 1 Department of Rehabilitation, Université Laval, Québec, Canada; 2 Centre interdisciplinaire de recherche en réadaptation et intégration sociale (CIRRIS), Centre Intégré universitaire de santé et de services sociaux de la Capitale-Nationale, Québec, Canada; 3 Faculty of Business Administration, Université Laval, Québec, Canada; 4 Centre interuniversitaire de recherche sur les réseaux d’entreprise, la logistique et le transport (CIRRELT), Montréal, Canada; 5 Faculty of Nursing, Université Laval, Québec, Canada; 6 Centre de recherche du CHU de Québec, Québec, Canada; 7 Department of Mechanical Engineering, Université Laval, Québec, Canada; 8 Centre de recherche en gestion des services de santé, Université Laval, Québec, Canada; 9 Department of Logistics and Operations Management, HEC Montréal, Montréal, Canada

**Keywords:** Patient prioritization, Systematic review, Healthcare services, Waiting lists, Outcomes

## Abstract

**Background:**

Waiting lists should be managed as fairly as possible to ensure that patients with greater or more urgent needs receive services first. Patient prioritization refers to the process of ranking referrals in a certain order based on various criteria with the aim of improving fairness and equity in the delivery of care. Despite the widespread use of patient prioritization tools (PPTs) in healthcare services, the existing literature on this subject has mainly focused on emergency settings. Evidence has not been synthesized with respect to all the non-emergency services.

**Methods:**

This review aims to perform a systematic synthesis of published evidence concerning (1) prioritization tools’ characteristics, (2) their metrological properties, and (3) their effect measures across non-emergency services. Five electronic databases will be searched (Cochrane Library, Ovid/MEDLINE, Embase, Web of Science, and CINAHL). Eligibility criteria guiding data selection will be (1) qualitative, quantitative, or mixed methods empirical studies; (2) patient prioritization in any non-emergency setting; and (3) discussing characteristic, metrological properties, or effect measures. Data will be sought to report tool’s format, description, population, setting, purpose, criteria, developer, metrological properties, and outcome measures. Two reviewers will independently screen, select, and extract data. Data will be synthesized with sequential exploratory design method. We will use the Mixed Methods Appraisal Tool (MMAT) to assess the quality of articles included in the review.

**Discussion:**

This systematic review will provide much-needed knowledge regarding patient prioritization tools. The results will benefit clinicians, decision-makers, and researchers by giving them a better understanding of the methods used to prioritize patients in clinical settings.

**Systematic review registration:**

PROSPERO CRD42018107205

**Electronic supplementary material:**

The online version of this article (10.1186/s13643-019-0992-x) contains supplementary material, which is available to authorized users.

## Background

In many healthcare settings, waiting lists help streamline the uncertain flow of requests and providers’ workload. Their presence in healthcare services is of paramount importance in order to rationalize capacity and hence costs [1]. However, as the demand for services exceeds supply, these lists can grow quickly and result in unacceptable wait times. Patients from countries with publicly funded healthcare systems frequently experience excessive wait times [2] that have significant consequences, such as a negative impact on pain and function and deterioration in the quality of life [3, 4]. Reducing wait times in order to improve access to healthcare services has become one of the main concerns of modern healthcare systems. Many strategies can be used to manage waiting lists with the aim of improving patient flow and reducing wait times. Examples of such approaches include increasing capacity (e.g., hiring more staff or purchasing more equipment), setting maximum wait time targets, and reorganizing patterns of services (e.g., introducing methods to reduce missed appointments) [1, 5]. However, wait times for non-urgent health services such as elective surgery or the prescription of wheelchairs is often unavoidable [6]. Therefore, waiting lists should be managed as fairly as possible to ensure that patients with greater or more urgent needs receive services ahead of those with less need and that patients with approximately the same degree of need wait about the same length of time [7]. Here, the concept of need goes beyond the medical perspective and incorporates wider social and environmental determinants associated with a sustainable vision of health [8]. Need in healthcare is commonly defined as the capacity to benefit. So, if health needs are present, then an effective intervention should be available to meet these needs and improve health [8].

One strategy to achieve fairness in managing waiting lists is to use patient prioritization methods. Patient prioritization refers to the process of ranking referrals in a certain order based on various criteria [9]. However, there are conflicting evidence regarding their effect on wait times [10]. Since the aim is for patients with the greatest needs to be treated first, patients’ wait times should be determined objectively on the basis of explicit and equitable criteria [2].

One challenge associated with patient prioritization is to compare patients’ priority dynamically based on their conditions and the amount of benefit expected from the services they are waiting for [7]. Thus, assessing patients’ priority tends to be inconsistent in practice and assessors do not agree on criteria [11, 12]. The lack of systematic and standardized practices for patient prioritization can have an impact on healthcare accessibility. For example, it has been found that “low priority” patients, such as those with chronic conditions, do not receive publicly accessible healthcare services in a timely manner because their condition is considered less urgent than others [13–15].

Several studies have investigated tools that could provide an explicit, transparent, and fair prioritization of patients on waiting lists. These tools focus on priority decisions in elective (i.e., medically necessary but non-emergency) settings where there is less likelihood of dealing with critical or life-threatening situations, as opposed to emergency services [16]. Instead, decisions may need to consider a range of factors with various degrees of importance for the main stakeholders (healthcare organizations, professionals, and patients and their families) [17–19]. These factors include pain, loss of function, risk of deterioration, impact of a disability on work or social roles, patient’s socioeconomic status, dependence on others, and economic impacts. Since many of these factors can be difficult to evaluate, patient prioritization is a complex task [20].

The purpose of patient prioritization tools (PPTs) is to help clinicians and decision-makers manage their programs or their institution’s waiting lists. These tools mainly comprise sets of general criteria that encompass personal factors (e.g., age), social factors (e.g., ability to work) [2], and any other factor deemed relevant (e.g., patient’s quality of life) [7, 21]. In addition, some tools are using specific criteria, which are more objective and measurable because they relate to disease-specific outcomes (e.g., visual acuity for cataract surgery) [21]. Most tools are designed for elective interventions, including cataract surgery [22], hip and knee arthroplasty [23, 24], and general surgery [25]. Similar tools have also been developed for prioritization in other non-emergency services, such as magnetic resonance imaging [26], children’s mental health services [27], and rehabilitation programs [5, 28].

PPTs can take different forms, including less formalized methods using a two- to four-level classification system (“high priority” and “low priority”), or more formal tools, which are most often priority scoring systems that assign a score to the needs of each patient [2]. There are some systems that prioritize informally based on clinical judgment, without using an explicit written tool [28]. In a recent study conducted in outpatient physiotherapy departments, stakeholders noted that the criteria used to prioritize patients were often vague and expressed a need for more explicit and objective criteria [29]. In-house prioritization tools were mostly developed through consultations and discussions with stakeholders in the program [28]. In contrast, several studies [22, 23, 25–27] present an iterative approach applied in different contexts to develop priority criteria based on a literature review and consultations with experts.

Many studies have tried to demonstrate the metrological properties of PPTs in healthcare services, such as reliability and validity [9, 11, 12, 22, 23, 25–27, 30]. In patient priority assessments, reliability refers to the interrater agreement (between two assessors) and intrarater stability (test-retest). In different contexts, studies have shown only poor to good interrater and test-retest reliability [22, 23, 25–27]. This means that independent assessors are likely to make different decisions regarding the rank of the same patient on the waiting list, and the same assessor may even make different decisions over time.

Validity refers to the extent to which an assessment measures what it is intended to measure and whether useful inferences can be made based on that measurement [31]. In the case of PPTs, a good demonstration of validity refers to patients who are rated as “high priority” and actually have the most needs [11, 31]. Validating PPTs is complicated by the lack of a gold standard with which to compare them [9, 11, 31]. On the other hand, construct validity can be tested by comparing them to other measurements which should measure similar dimensions in a consistent way [11]. For example, Escobar et al. correlated scores between a prioritization tool and Western Ontario McMaster Osteoarthritis Index (WOMAC) questionnaire dimensions (quality of life measurement) to determine its validity [31]. They found that those with poorer WOMAC scores have a tendency to score higher on this prioritization tool, which supports the validity of this tool to be used with patients waiting for hip or knee replacement [31].

PPTs have already been the subject of two systematic reviews, but authors limited their search to allied health services [11] or elective surgeries [21]. MacCormick et al. reviewed evidence published before 2001 about prioritization for elective surgery by focusing on different ethical-based prioritization, what different criteria are in use, how they were derived, and what different tools of summation are in use [21]. Harding et al., meanwhile, have sought to determine the properties of triage systems for allied health services, including emergency setting, and the health services’ outcomes [11]. They tried to synthesize evidence of outcome measures related to patient flow (e.g., waiting times, effect on waiting lists, and length of stay) but found that PPTs’ effectiveness is challenging to prove [10]. In addition to limited evidence, studies in this review were mostly conducted in emergency settings. Measuring outcomes of a PPT needs to be documented in a variety of settings and to take different perspectives into account, including those of the professionals, the system, and the service users [11].

Despite the widespread use of PPTs in non-emergency healthcare services, the existing literature on this subject has not been synthesized. Indeed, there is little evidence regarding the current status of PPTs in healthcare systems. It is crucial to build on a synthesis of the properties of PPTs across healthcare services in order to have a better understanding of this innovation that aims to optimize service efficiency and access to patient care. This review aims to systematically synthesize the published evidence concerning PPTs across non-emergency settings. Our objectives are to (1) describe PPTs’ characteristics, such as format, description, population, setting, purpose, criteria, and developer; (2) identify their metrological properties, such as reliability and validity; and (3) point out their effect or outcome measures (e.g., decrease of wait times). The research question of this systematic review is: “What is the status of PPTs in healthcare systems in terms of characteristics, metrological properties and effect measures?”

## Methods

A mixed studies review will be carried out to have a better understanding of this complex intervention [32]. This review follows stages proposed by Pluye and Hong [32], guiding systematic reviews and types of mixed studies synthesis designs. The present protocol has been registered within the PROSPERO database (registration number: CRD42018107205) and is being reported in accordance with the Preferred Reporting Items for Systematic Reviews and Meta-Analyses Protocols (PRISMA-P) statement [33, 34] (see PRISMA-P checklist in Additional file 1).

### Search strategy

The research question was formulated using the ECLIPSE model (Expectation, Client Group, Location, Impact, Professionals, Service) to guide the systematic review, which is generally used in systematic reviews in the field of health and social services management [35]. It offers an alternative to the PICO framework that is mostly used in systematic reviews of clinical interventions but is less applicable in the domain of management. Two of the authors (JD and MEL) will determine and validate the keywords and inclusion/exclusion criteria guiding the systematic review in accordance with its objectives. They will evaluate the quality of the search strategy using the Peer Review of Electronic Search Strategies Evidence-Based Checklist (PRESS EBC) criteria [36, 37].

A professional librarian will initiate the database research using free-text terms, Boolean operators, and truncation. An example of the search strategy developed for MEDLINE/Ovid is provided in the Appendix (Table 1). This search strategy will be adapted for each database.

### Information sources

We will search in five electronic databases (Cochrane Library, Ovid Medline, Embase, Web of Science, and CINAHL) using the search strategy developed by a team of researchers and a professional librarian. Then, to identify additional sources, a secondary four-step search will be conducted: (1) screening of the reference lists of the identified articles; (2) citation searches performed using Google Scholar for records that meet the inclusion criteria; (3) screening of 25 similar references suggested by the databases, where available; and (4) contact authors who have published two articles or more that are included in our review. Data will not be limited by timeframe, and we will consider all studies published before September 2018.

### Eligibility criteria

The inclusion criteria for the selection of articles in the literature review will be (1) peer-reviewed quantitative/qualitative/mixed methods empirical studies, which includes all qualitative research methods (i.e., phenomenological, ethnographic, grounded theory, case study, and narrative) and quantitative research designs (i.e., randomized controlled studies, cohort studies, case-control, cross-sectional, descriptive, and longitudinal); (2) published in English or French before September 2018; (3) reporting the use of a PPT in a non-emergency healthcare setting; and (4) discussing characteristics, metrological properties, or effect measures of a PPT.

The following types of references will be excluded from the literature review: (1) studies focusing on strategies/methods for managing waiting lists without using a prioritization tool or system, (2) studies conducted in an emergency setting, (3) articles about healthcare systems dealing with critical or life-threatening situations (e.g., organ transplants), and (4) literature reviews.

### Data management

Two reviewers (JD and SD) will import all records from the database in a reference management software (Endnote). Duplicates will be removed using the software command “Find duplicates” based on the title of the references. The remaining duplicates will be deleted during the abstract/title screening process.

### Screening and selection process

The same two reviewers will independently screen all articles identified from the search. First, titles and abstracts of articles returned from initial searches will be screened based on the eligibility criteria outlined above. At this point, we estimate to select 50 to 75 potentially relevant records. Second, full texts will be examined in detail and screened for eligibility. We anticipate to find 10 to 20 relevant studies meeting all eligibility criteria. Third, references of all considered articles will be hand-searched to identify any relevant report missed in the search strategy by two reviewers independently. Any disagreement between reviewers will be resolved by discussion to meet a consensus. In case of disagreements concerning the selection of articles, a third reviewer (MEL) will evaluate the eligibility of the studies concerned. We will measure the agreement between reviewers with the Kappa coefficient.

### Data extraction

Independently, two reviewers (JD and SD) will review all articles in the final list using an extraction grid. The extraction grid will include items such as information relating to the authors, title of publication, year of publication, level of evidence, and information about the PPT. Data will be sought to itemize format, description, population, setting, purpose, criteria, developer, metrological properties, and outcome measures. All these variables have been used to report data in former systematic reviews and will help to meet our review objectives as well. Additional information including authors’ key conclusions and other relevant findings will also be extracted.

### Data synthesis

A sequential exploratory synthesis design method will be used to describe the results of the systematic mixed studies review [32]. This synthesis method is performed in two steps, where in phase one, qualitative data from qualitative, quantitative, and mixed studies are transformed into qualitative findings (i.e., a taxonomy of study results) using qualitative thematic analysis, for instance. At this step, we will follow an inductive thematic analysis of data and a computer-assisted qualitative data analysis software could be used (i.e., NVivo). In phase two, quantitative data from quantitative studies and mixed studies are tabulated and compared when there is a common variable across studies (i.e., a common type of study results) [38]. This sequential exploratory synthesis method is appropriate and complete in itself because we can use qualitative studies to interpret, explain, or provide more insight to some quantitative findings [39].

### Critical appraisal

Two independent assessors (JD and SD) will assess the methodological quality of the studies. Pluye et al.’s MMAT for appraising the methodological quality of studies with diverse designs [40] will be used to evaluate the quality of the articles. This tool is appropriate for our review because it contains specific criteria to assess the methodological quality of mixed methods studies. It allows the use of only one tool for concomitantly appraising all types of empirical studies, whether quantitative or qualitative [40]. As proposed, we will discuss the strength of the body of evidence by a detailed presentation of the ratings of each criterion to better inform the quality of the included studies [41].

## Discussion

In a world where the demand for efficient and equitable health services is growing substantially, it is of utmost importance to optimize healthcare service resources and waiting list management [2]. This systematic review will provide much-needed knowledge regarding PPTs. The results will benefit clinicians, decision-makers, and researchers by giving them a better understanding of the methods used to prioritize patients in clinical settings.

To our knowledge, there are no guidelines about tools that help clinicians and healthcare managers to make decisions about patient prioritization in non-emergency settings. This study will lead to a synthesis of these practices and a better understanding of the various prioritization methods used in healthcare organizations. A common vocabulary and clear guidelines are needed to show characteristics of PPTs, how they are developed, and how to measure their effects. Ultimately, this knowledge synthesis is expected to benefit a large population because this knowledge could be disseminated across different organizations. Knowledge translation activities could inform stakeholders about PPTs and how they could be adapted for their programs or services. We could, for example, organize workshops with decision-makers and clinicians aiming to formalize their prioritization practice according to relevant findings from this literature review.

We anticipate potential limitations in this study. First, the concept of “tool” is not well defined in the literature, which could make it difficult to identify all relevant studies using a PPT. Second, prioritization is a complex intervention mixing a wide variety of domains, such as engineering, public health, and management, so it would be challenging to synthesize data from all these fields. Third, triage and prioritization are often used as interchangeable terms, which could compromise the relevance of studies included in the review, because we want to focus precisely on PPTs. All important protocol amendments will be documented in a logbook and they will be clearly mentioned in the method section of the systematic review publication.

### Additional file

Additional file 1: PRISMA-P 2015 Checklist. PRISMA-P checklist adapted for use with systematic review protocol submissions to BioMed Central journals. (DOCX 32 kb)

## Appendix

**Table 1:** Example of search strategy in MEDLINE/Ovid database

#	Search
1	(client* or patient* or “service user*”).ti. or (client* or patient* or “service user*”).ab.
2	Patient Selection.sh.
*3*	*1 or 2*
4	(categor* or setting or strateg* or system* or tool*).ti. or (categor* or setting or strateg* or system* or tool*).ab.
5	Classification/
*6*	*4 or 5*
7	(queu* or delay* or “waiting time*” or “wait time*” or “waiting list*” or “wait list*” or waitlist*).ti. or (queu* or delay* or “waiting time*” or “wait time*” or “waiting list*” or “wait list*” or waitlist*).ab.
8	Systems Theory.sh.
9	Waiting Lists.sh.
*10*	*7 or 8 or 9*
11	(priorit* or triag*).ti. or (priorit* or triag*).ab.
12	Health Priorities.sh.
13	Triage.sh.
*14*	*11 or 12 or 13*
15	(“health service*” or “healthcare service*”).ti. or (“health service*” or “healthcare service*”).ab.
16	Health Services.sh.
*17*	*15 or 16*
*18*	*3 or 6 or 10*
19	*14 and 18*
20	*17 and 19*

## Abbreviations

CINAHL: Cumulative Index to Nursing and Allied Health Literature
PPT: Patient prioritization tool
PRESS EBC: Peer Review of Electronic Search Strategies Evidence-Based Checklist
PRISMA: Preferred Reporting Items for Systematic Reviews and Meta-Analyses
